# Factors Influencing Domestic Human Trafficking in Africa: Protocol for a Scoping Review

**DOI:** 10.2196/56392

**Published:** 2024-11-18

**Authors:** Loubna Belaid, Ivan Sarmiento, Anna Dion, Andrés Rojas Cardenas, Anne Cockcroft, Neil Andersson

**Affiliations:** 1 École Nationale d'Administration Publique Montreal, QC Canada; 2 Department of Family Medcine (CIET/PRAM) McGill University Montreal, QC Canada; 3 Department of Family Medicine (PRAM) Montreal, QC Canada; 4 Department of Family Medicine (CIET/PRAM) McGill University Montreal, QC Canada

**Keywords:** domestic human trafficking, Africa, scoping review protocol, protocol, human trafficking, trafficking, modern slavery, human rights violation, discourse analysis, exploitation, fuzzy cognitive map

## Abstract

**Background:**

Human trafficking is a human rights violation in every region of the world. The African continent is not spared. Every year, millions of people experience significant health and social consequences. International organizations and governments combating human trafficking are hindered by a lack of knowledge about what factors influence domestic (within-country) human trafficking.

**Objective:**

This study aims to conduct a scoping review to collate and synthesize literature on factors influencing domestic trafficking in Africa.

**Methods:**

We will follow Arksey and O’Malley’s framework to answer the question about reported influences on domestic human trafficking and their relative weight. The search strategy will explore PubMed, CINAHL, Web of Science, and Scopus. A total of 2 independent researchers will select quantitative, qualitative, or mixed methods studies that examine relationships influencing domestic human trafficking. We will document our results by following the PRISMA-ScR (Preferred Reporting Items for Systematic Reviews and Meta-Analyses extension for Scoping Reviews) guidelines. We will extract a list of all reported relationships between identified factors influencing domestic human trafficking in each study. Based on a discourse analysis approach, we will weigh the strengths of the relationships based on how frequently they are reported across the included studies. We will summarize the findings as fuzzy cognitive maps depicting the relationships reported in the literature. The maps represent the influences between concepts (nodes) linked by arrows (edges) going from each cause to its outcomes. These maps are helpful visual summaries of the factors associated with domestic human trafficking, allowing a comparison with maps to be created by stakeholder groups.

**Results:**

This project received financial support in March 2023. We expect to start the project in March 2024. We recruited 2 research staff members to conduct the scoping review and expect to publish the results in March 2025.

**Conclusions:**

The review will provide a comprehensive understanding of factors influencing domestic human trafficking in Africa. The overlap of human trafficking with other forms of exploitation, the limited literature on domestic human trafficking, and the likely diversity of factors are challenges for the review. We propose strategies to address these challenges.

**International Registered Report Identifier (IRRID):**

PRR1-10.2196/56392

## Introduction

Human trafficking, also known as modern slavery, is a human rights violation. Every year, millions of people experience significant health and social consequences. In 2021, the Global Slavery Index estimated that 50 million people were living in slavery, an increase of 10 million since 2016 [1]. In Africa, 7 million men, women, and children were living in modern slavery, a prevalence of 5.2 people for every 1000 people [2].

“Human trafficking,” “Modern slavery,” and “Trafficking in person” are umbrella terms to refer to both sex trafficking and forced labor [3,4]. The 2000 United Nations Palermo Protocol defines human trafficking as follows:

The recruitment, transportation, transfer, harbouring or receipt of persons, by means of the threat or use of force or other forms of coercion, of abduction, of fraud, of deception, of the abuse of power or of a position of vulnerability or of the giving or receiving of payments or benefits to achieve the consent of a person having control over another person, for the purpose of exploitation [5].

Human trafficking can include but does not require movements of people. People may be considered trafficking victims regardless of whether they were born into a state of servitude, were exploited in their hometown, or were transported to the exploitative situation [3].

In domestic human trafficking situations, all stages of the trafficking process, including the recruitment of victims, occur within national borders [6].

International human trafficking has received more attention compared with domestic human trafficking [7-10], even though most people are trafficked within their home countries; a 2022 United Nations report mentioned that of the people trafficked in sub-Saharan Africa that are detected, 85% are domestically trafficked [11].

Evidence on factors influencing human trafficking in Africa is emerging. A scoping review of domestic and international human trafficking in Ethiopia found that push factors of human trafficking are poverty, gender discriminatory practices, environmental factors, poor governance, demographic factors, famine, war, political instability, and economics. The pull factors are the demand for domestic workers and cheap uncompensated labor [12]. In the same review, sociocultural factors were identified as factors leaving women and children vulnerable to trafficking: early child marriage, lack of access to social services in rural areas, limited access to education (especially for girls), limited parental education, family discord, dissatisfaction with traditional ways of life, the attraction of paid work to support natal families, pregnancy outside of marriage and associated stigma, leaving school at an early age, and large family size [12].

A 2022 mixed methods study among women and girls trafficked in Uganda and Nigeria reported that family poverty, violence, and neglect exposed them to being trafficked nationally and internationally [13]. The participants described traumatic events in their family lives, such as the loss of one or both parents, emotional abuse, physical and sexual violence by caregivers, neglect, sexual exploitation, child labor, witnessing violence, parental alcohol abuse, parental divorce, extreme poverty, and hunger before being trafficked [13]. A 2023 qualitative study among youth trafficked nationally and internationally in Uganda identified poverty and abusive home life, frequently triggered by the death of a caretaker. Limited education, lack of social support, and survival needs pushed people who are trafficked into exploitative situations [14]. The available evidence does not give a clear picture of the specific factors influencing domestic trafficking.

In addition, the antitrafficking responses are not sufficiently aligned with the contexts in which they are implemented [15]. A 2024 systematic review on implementation science research in exit and postexit human trafficking interventions in sub-Saharan Africa reported on how local perspectives on poverty, gender norms, child labor, and family expectations differ from implementer’s perspectives (mostly outsiders), which undermined social acceptability and potential effectiveness of anti–human trafficking strategies [15].

While there is emerging evidence on factors associated with human trafficking in Africa, there is a critical need to expand our understanding of factors influencing specifically domestic human trafficking to improve co-design interventions to combat human trafficking [16]. Developing effective evidence-based strategies requires participatory approaches that allow stakeholders to contextualize the available literature and discuss the most effective strategy locally [17]. This scoping review will collate and synthesize the published literature on factors influencing domestic human trafficking in Africa to inform evidence-based policies and guide future research efforts.

## Methods

### Study Design

**Figure 1:**
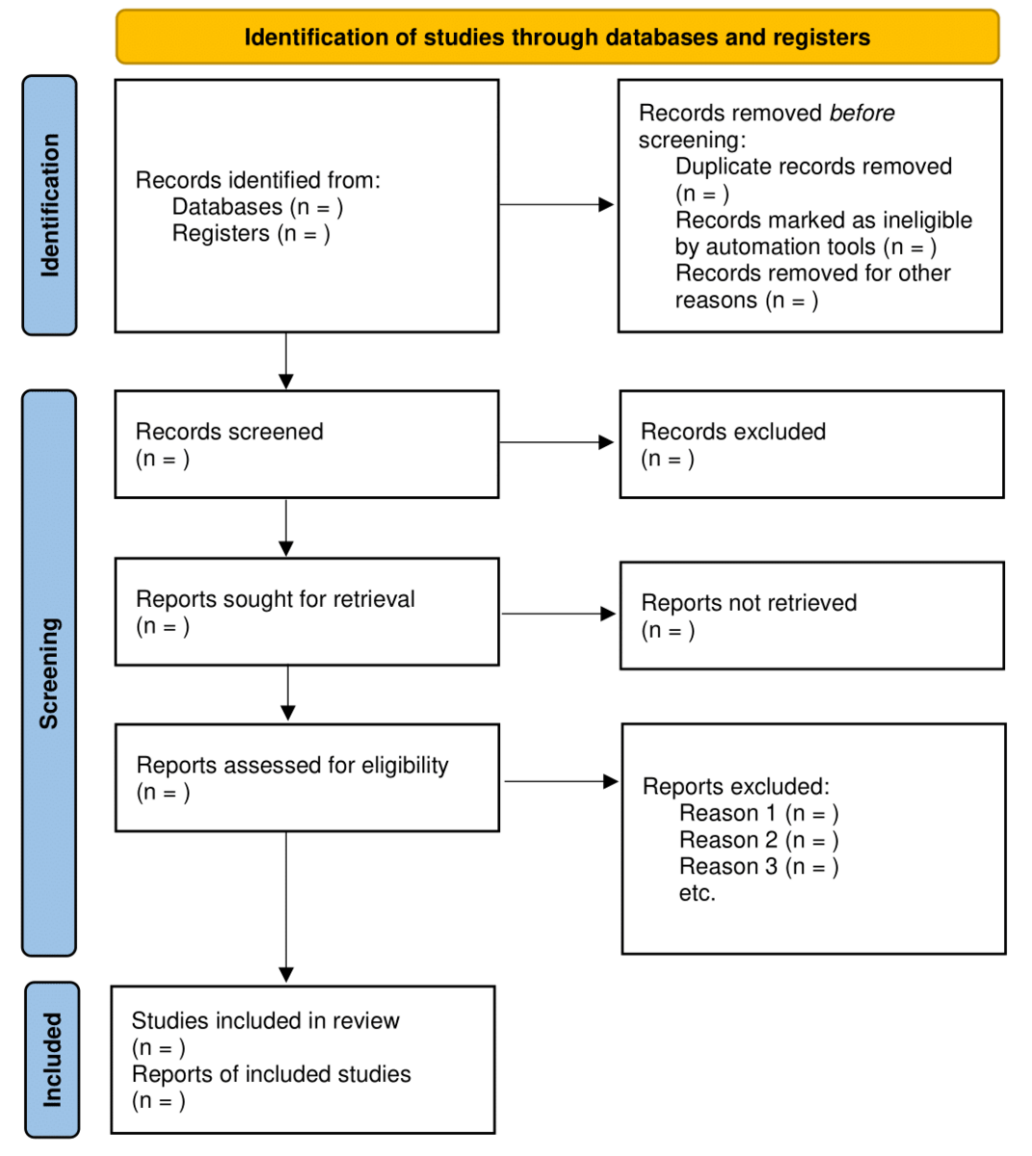
PRISMA (Preferred Reporting Items for Systematic Reviews and Meta-Analyses) flowchart template. For the “Records identified from” section, we will report the number of records identified from each database or register searched (rather than the total number across all databases or registers). For the “Records excluded” section, if automation tools were used, we will indicate how many records were excluded by a human and how many were excluded by automation tools.

This scoping review will follow the Arksey and O’Malley [18] framework for scoping reviews and use the PRISMA-ScR (Preferred Reporting Items for Systematic Reviews and Meta-Analyses extension for Scoping Reviews) guidelines to report the review [19] (Figure 1 [20] and Multimedia Appendix 1).

Scoping studies are knowledge syntheses that “aim to map rapidly the key concepts underpinning a research area and the main sources and types of evidence available and can be undertaken as stand-alone projects, especially where an area is complex or has not been reviewed comprehensively before” [18]. Arksey and O’Malley [18] developed an approach to scoping review based on six stages: (1) identifying a research question; (2) identifying relevant studies; (3) study selection; (4) charting data; (5) summarizing, synthesizing, and reporting the results; and (6) consultation.

### Stages

#### Stage 1: Identification of Research Question

The primary research questions are (1) What factors influence domestic human trafficking in Africa? and (2) Based on the available evidence, what is the relative weight of their influence?

The secondary research questions are (1) What methods have researchers used to identify factors influencing domestic human trafficking? and (2) What are the research gaps on factors influencing domestic human trafficking?

#### Stage 2: Identifying Relevant Studies

With the help of a specialist librarian, we will design a search strategy using Medical Subject Headings terms and Boolean operators (“AND” and “OR”; Multimedia Appendix 2). We will search the electronic databases such as PubMed, CINAHL, Web of Science, and Scopus and manually search the reference lists of included articles.

The review will include peer-reviewed articles on human populations reported in English and French. We will restrict geographical settings to Africa and focus on the last 24 years of publication (2000 to 2024). In 2000, the United Nations Convention against Transnational Organized Crime adopted a protocol to prevent, suppress, and punish trafficking in persons, with a focus on women and children. The protocol defines the crime of trafficking in human beings [5].

This scoping review will focus on domestic human trafficking, as defined in the *Introduction* section:

All the stages of the trafficking process occur within a country’s borders. There are no international cross-border movements. However, victims can be moved across or within cities or provinces [6].

This form of trafficking can involve various types of exploitation, including forced labor, sexual exploitation, forced marriage, and other forms of abuse and coercion [3,4].

In this review, we will use the terms human trafficking and modern slavery interchangeably if it refers to “situations of exploitation that a person cannot refuse or leave because of threats, violence, coercion, deception, and/or abuse of power” [3,4].

The strategy for identifying relevant articles has 2 steps. The first is an initial search in MEDLINE and PubMed to analyze text words in titles, abstracts, and index terms. The second step updates the term and runs the search strategies in all databases.

#### Stage 3: Study Selection

After completing the search and excluding duplicated records, we will export the list of references into Covidence to support the selection process [21]. A total of 2 independent researchers will select the studies. They will screen titles and abstracts against the predetermined inclusion and exclusion criteria to identify potentially relevant papers. A second round will use the full papers to support the selection decision. An independent team member will reconcile differences between the reviewers if they occur.

To be included, a paper must (1) be a peer-reviewed paper; (2) include human participants (women, men, and children) who self-identified or were defined by researchers as having been trafficked or are in a trafficked situation; (3) research takes place in African countries; (4) focus on domestic human trafficking, forced labor (domestic servitude, factory, mine, agriculture, and fishing industries), or sexual exploitation, forced marriage, and forced begging; (5) be an empirical study using qualitative, quantitative, or mixed methods analysis; and (6) report factors influencing domestic human trafficking. By factor, we mean circumstances and situations perceived as a cause of domestic human trafficking. The protocol will not focus on a specific type of participants (survivors, traffickers, etc) or levels (micro, meso, and macro) of factors. We will consider all of them and coherently organize them in charting and synthesizing data stages.

The exclusion criteria are (1) papers about exclusively international human trafficking, which refer to foreigners (often from countries with limited economic resources) who are brought to another country for labor or sex exploitation; (2) protocols; (3) theoretical, conceptual, and discussion papers; (4) commentaries, conference abstracts, letters of correspondence, and editorials; (5) PhD and master’s dissertations; (6) papers about animal trafficking; (7) papers about organ trafficking; (8) research that takes place only in Europe, North America, and Asian countries; and (9) papers published before 2000. Scoping review methods do not require an assessment of the quality of evidence [19].

#### Stage 4: Charting Data

Based on the reading of full texts, 2 independent reviewers will extract the following items into Covidence:

1. Study characteristics (title, year of publication, country, study design, population, and sample size)
2. Type of trafficking (labor, sexual exploitation, domestic servitude, and forced marriage)
3. Scope of the study (national, regional, provincial, districts, municipality, and villages)
4. Settings and units of analysis (nongovernmental organizations, community-based or faith organizations, health services, and schools)
5. Methods to identify and assess factors influencing human trafficking
6. Strategies for participant recruitment
7. Relationships describing associations with domestic human trafficking are described with three elements: (A) factors that influence an outcome, (B) the outcome of the relationship, and (C) a sign to indicate if the relationship is positive or negative
8. Contextual data (eg, law enforcement, legal framework, antitrafficking local policies, statistics of domestic human trafficking, survivor’s profiles, and the type of human trafficking more prevalent)

A total of 2 reviewers will pilot the data extraction using a random sample of 5 articles. They will use an Airtable application to chart the data. The pilot will refine the data extraction template and ensure the consistency of the extraction process across reviewers.

#### Stage 5: Summarize, Synthesize, and Report the Results

Reporting will include tables and charts of the geographic and population distribution of studies, methods used, and findings about the factors influencing domestic trafficking.

**Figure 2:**
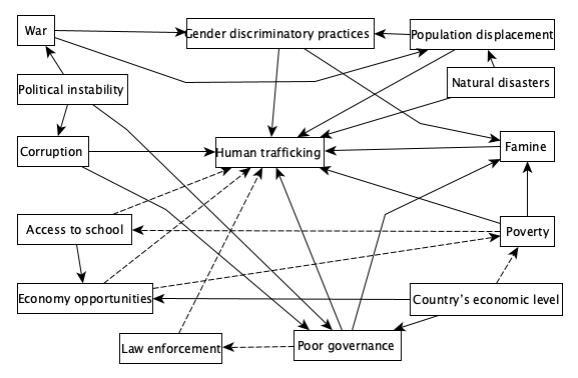
An example of a fuzzy cognitive map.

We will depict the scoping review findings as a fuzzy cognitive map reflecting the reported factors influencing domestic trafficking. Fuzzy cognitive mapping can combine diverse knowledge sources to inform research and actions [22]. The maps depict factors (influences) as nodes linked by weighted arrows (relationships and the strength of their influence) [23,24]. We will identify the reported influences with outcomes for each study and indicate if they are positive (+1) or negative (–1). A relationship is positive if the increment of the origin factor results in the outcome’s increment (an increased risk of trafficking). If an increase in the influence results in a decrease in the outcomes, the relationship is negative (a reduction of trafficking). Figure 2 shows hypothetical influences on domestic trafficking to present an example of a fuzzy cognitive map. A direct relationship will have a direct arrow from one concept (factor) to the primary outcome (human trafficking). In Figure 2, famine increases the risk of domestic human trafficking. An indirect relationship will have an arrow from one to more concepts before reaching the outcome. For instance, political instability leads to war, which in turn increases gender discriminatory practices, which in turn leads to an increase in human trafficking. Arrows in dotted lines represent relationships of protective factors associated with human trafficking. For example, more economic opportunities lead to less poverty. More access to school leads to less human trafficking. This technique helps to identify all the direct and indirect relationships influencing an outcome and the nature of the relationships, which are either positive (increase the risk of being trafficked) or negative (decrease the risk of being trafficked).

We will calculate fuzzy transitive closure on the collection of relationships from each study, identifying the maximum influence one cause can have on other outcomes through direct and indirect connections [24,25]. Based on a discourse analysis approach, we will weigh the relationships’ strengths based on how frequently they are reported across the included studies [26]. A relationship reported by many studies in the literature would have a more substantial weight than a relationship reported in only 1 or 2 studies. We will count the number of times a relationship appears in the literature and divide this number by the maximum frequency. Values closer to 1 will have more weight in the literature, and values closer to 0 will have less weight.

#### Stage 6: Consultations

This scoping review is a step in a proposed more extensive participatory research project on domestic human trafficking in an African setting. We will use the literature map from this scoping review to compare with the stakeholder maps we plan to create [22]. Engaging stakeholders in the process is essential because it will allow them to consider the available research evidence in the context of their views and support them in making evidence-informed decisions [17].

The stakeholders are domestic human trafficking survivors, community leaders and service providers, and representatives from relevant government ministries and nongovernmental organizations.

We will invite each stakeholder group separately to join a fuzzy cognitive mapping session. With the support of 2 facilitators (facilitator and notetaker), each stakeholder group will draw a map.

Fuzzy cognitive maps are soft models representing perceived causality about an issue. They describe the factors that stakeholders consider to be the causes of an outcome [22,23].

In this case, stakeholders will identify perceived causal factors influencing domestic human trafficking. During a mapping session, the facilitator writes the outcome (domestic human trafficking) on a magnetic tile of a whiteboard. The facilitator invites participants to mention the causes of the outcome, writing each cause on a tile and placing it on the board. Then, the facilitator asks participants to identify relationships between factors, and the facilitator draws arrows between them. The facilitator invites participants to determine which one is the strongest (weight 5) and which one is the weakest (weight 1) arrow. The participants assign weights to all the other relationships (weights 2-4). When the map is complete, the facilitator takes a picture of the map. We have used this technique successfully in many projects [27-30].

We will digitize the maps with yEd (yWorks GmbH) software that generates, imports, and manages diagrams [31]. We will undertake fuzzy transitive closure to determine all possible indirect relationships between factors as part of a longer causal pathway of connected factors [25]. Social network analysis will identify the digitized maps’ most influential perceived causes and critical intermediate outcomes [23]. Stakeholders will compare the map based on the scoping review with their own maps of perceived causes of domestic human trafficking. The scoping review and stakeholder maps will provide the evidential base for deliberative dialogue.

## Results

This project received financial support in March 2023. We expect to start this project in March 2024. We recruited 2 research staff to conduct the scoping review. We expect to publish the scoping review results in March 2025.

## Discussion

### Expected Findings

This scoping review will collate and synthesize the published literature on factors influencing domestic human trafficking in Africa.

The review will highlight the relationships associated with domestic human trafficking, allowing researchers and practitioners to focus on them to design and target their interventions, potentially increasing their effectiveness. The review will determine the most influential “pathways,” from potential causes to the outcome of domestic human trafficking. It will identify gaps in the evidence and guide future research.

Human trafficking involves interconnected social, geographic, health, legal, new technologies (artificial intelligence and social media), political, and economic factors. Therefore, the scoping review findings could be relevant to researchers and practitioners from many disciplines.

We expect some challenges in conducting the review. The published literature overlaps the concept of human trafficking with other concepts, such as smuggling and internal migration. Few studies might be conducted on domestic human trafficking. Much domestic human trafficking occurs underground and is unrecognized and underreported. To ensure we effectively capture studies of domestic human trafficking, we will adjust the search strategy based on the text of the initially identified articles. We will document changes to the initial search matrix.

The scoping review may reveal a wide range of relationships reported in the literature, making it difficult to identify the most influential ones. The transitive closure of the fuzzy cognitive maps from the scoping review will help objectively determine the most influential “pathways” from potential causes to the outcome of domestic human trafficking [25,32].

This review will inform the design of evidence-based policies and guide future research efforts.

### Conclusions

Research on human trafficking in Africa is growing; this review will collate all the currently available evidence on factors influencing domestic trafficking. It will inform evidence-based policies and guide future research efforts.

## Appendix

Multimedia Appendix 1 PRISMA flow diagram.

Multimedia Appendix 2 Search strategies in electronic databases.

## Abbreviations

PRISMA-ScR: Preferred Reporting Items for Systematic Reviews and Meta-Analyses extension for Scoping Reviews

## Data Availability

Data sharing does not apply to this paper as no data sets were generated or analyzed during this study.
