# Supplementary material for: Factors Influencing Domestic Human Trafficking in Africa: Protocol for a Scoping Review
Source: JMIR Res Protoc. 2024 Nov 18;13:e56392. doi: 10.2196/56392 (PMC11612573; doi:10.2196/56392)
Supplement: Multimedia Appendix 2 [file resprot_v13i1e56392_app2.docx]

Appendix 1: Search strategies in electronic databases

| PubMed  (264 results) | ((("Human Trafficking"[Mesh] OR "trafficking in person*" [tw]) OR (("sexual traffic*"[tiab] OR "sex* exploitation" [tiab] OR "sex* slave*"[tiab] OR prostitut* [tiab] OR "forced prostitut*"[tiab] OR "forced marriag*"[tiab]))) OR (("labor traffic*" [tiab] OR "labour traffic*" [tiab] OR "forced lab*"[tiab] OR "labor exploitation*" [tiab] OR "labour exploitation*" [tiab] OR "debt bondage"[tiab] OR "bonded lab*" [tiab] OR "modern slave*" [tiab] OR "domestic servitude" [tiab]))) AND (Africa OR Algeria [tiab] OR Angola [tiab] OR Benin [tiab] OR Botswana [tiab] OR "Burkina Faso" [tiab] OR Burundi [tiab] OR "Cabo Verde" [tiab] OR Cameroon [tiab] OR "Central African Republic" [tiab] OR Chad [tiab] OR Comoros [tiab]OR Congo [tiab] OR "Democratic Republic of the Congo" [tiab] OR "Cote d'Ivoire" [tiab] OR Djibouti [tiab] OR Egypt [tiab] OR "Equatorial Guinea" [tiab] OR Eritrea [tiab] OR Eswatini [tiab] OR Ethiopia [tiab] OR Gabon [tiab] OR Gambia [tiab] OR Ghana [tiab] OR Guinea [tiab] OR "Guinea-Bissau" [tiab] OR Kenya [tiab] OR Lesotho [tiab] OR Liberia [tiab] OR Libya [tiab] OR Madagascar [tiab] OR Malawi [tiab] OR Mali [tiab] OR Mauritania [tiab] OR Mauritius [tiab] OR Morocco [tiab] OR Mozambique [tiab] OR Namibia [tiab] OR Niger [tiab] OR Nigeria [tiab] OR Rwanda [tiab] OR "Sao Tome and Principe" [tiab] OR Senegal [tiab] OR Seychelles [tiab] OR "Sierra Leone" [tiab] OR Somalia [tiab] OR "South Africa" [tiab] OR "South Sudan" [tiab] OR Sudan [tiab] OR Tanzania [tiab] OR Togo [tiab] OR Tunisia [tiab] OR Uganda [tiab] OR Zambia [tiab] OR Zimbabwe [tiab] OR "Northern Africa*" [tiab] OR "Middle Africa*" [tiab] OR "Eastern Africa*" [tiab] OR "Western Africa*" [tiab]) AND (2000:2023[pdat]) |
| --- | --- |
| CINAHL (1068 results) | MH human trafficking OR TI ( trafficking in persons OR sex* traffic*” OR “sex* exploitation” OR “sex* slave*” OR prostitut* OR “forced prostitut*” OR “forced marriag*” OR “lab* traffic*” OR “forced lab*” OR “lab* exploitation*” OR “lab* coercion” OR “debt bondage” OR “bonded lab*” OR “modern slave*” OR “contemporary salve*” OR “domestic servitude” OR “child harvest*” ) OR AB ( trafficking in persons OR sex* traffic*” OR “sex* exploitation” OR “sex* slave*” OR prostitut* OR “forced prostitut*” OR “forced marriag*” OR “lab* traffic*” OR “forced lab*” OR “lab* exploitation*” OR “lab* coercion” OR “debt bondage” OR “bonded lab*” OR “modern slave*” OR “contemporary salve*” OR “domestic servitude” OR “child harvest*” ) AND TX ( Africa OR Algeria OR Angola OR Benin OR Botswana OR "Burkina Faso" OR Burundi OR "Cabo Verde" OR Cameroon OR "Central African Republic" OR Chad OR Comoros [tiab]OR Congo OR "Democratic Republic of the Congo" OR "Cote d'Ivoire" OR Djibouti OR Egypt OR "Equatorial Guinea" OR Eritrea OR Eswatini OR Ethiopia OR Gabon OR Gambia OR Ghana OR Guinea OR "Guinea-Bissau" OR Kenya OR Lesotho OR Liberia OR Libya OR Madagascar OR Malawi OR Mali OR Mauritania OR Mauritius OR Morocco OR Mozambique OR Namibia OR Niger OR Nigeria OR Rwanda OR "Sao Tome and Principe" OR Senegal OR Seychelles OR "Sierra Leone" OR Somalia OR "South Africa" OR "South Sudan" OR Sudan OR Tanzania OR Togo OR Tunisia OR Uganda OR Zambia OR Zimbabwe OR "Northern Africa*" OR "Middle Africa*" OR "Eastern Africa*" OR "Western Africa*" ) |
| Web of Science  (1230 results) | (((TS=("human trafficking")) OR TS=("trafficking in persons" OR "sex* traffick*” OR “sex* exploitation” OR “sex* slave*” OR prostitut* OR “forced prostitut*” OR “forced marriag*” OR “lab* traffick*” OR “forced lab*” OR “lab* exploitation*” OR “lab* coercion” OR “debt bondage” OR “bonded lab*” OR “modern slave*” OR “domestic servitude” OR “child harvest*” )) AND TS=(Africa OR Algeria OR Angola OR Benin OR Botswana OR "Burkina Faso" OR Burundi OR "Cabo Verde" OR Cameroon OR "Central African Republic" OR Chad OR Comoros [tiab]OR Congo OR "Democratic Republic of the Congo" OR "Cote d'Ivoire" OR Djibouti OR Egypt OR "Equatorial Guinea" OR Eritrea OR Eswatini OR Ethiopia OR Gabon OR Gambia OR Ghana OR Guinea OR "Guinea-Bissau" OR Kenya OR Lesotho OR Liberia OR Libya OR Madagascar OR Malawi OR Mali OR Mauritania OR Mauritius OR Morocco OR Mozambique OR Namibia OR Niger OR Nigeria OR Rwanda OR "Sao Tome and Principe" OR Senegal OR Seychelles OR "Sierra Leone" OR Somalia OR "South Africa" OR "South Sudan" OR Sudan OR Tanzania OR Togo OR Tunisia OR Uganda OR Zambia OR Zimbabwe OR "Northern Africa" OR "Middle Africa" OR "Eastern Africa" OR "Western Africa" )) AND (PY==("2023" OR "2022" OR "2021" OR "2020" OR "2019" OR "2018" OR "2017" OR "2016" OR "2015" OR "2014" OR "2013" OR "2012" OR "2011" OR "2010" OR "2009" OR "2008" OR "2007" OR "2006" OR "2005" OR "2004" OR "2003" OR "2002" OR "2001" OR "2000")) |
| Scopus  (2997 results) | TITLE-ABS-KEY ( "human traffic*" OR "traffic* in person*" OR ( modern OR contemporary ) AND slave* ) OR TITLE-ABS-KEY ( "sex* traffick*" OR "sex* exploitat*" OR "sex* slave*" OR prostitut* OR "forced prostitut*" OR "forced marriag*" ) OR TITLE-ABS-KEY ( "domestic servitude" OR "child harvest*" ) OR TITLE-ABS-KEY ( "lab* traffic*" OR "force* lab*" OR "lab* coerc*" OR "lab* exploit*" OR "lab manipulat*" ) AND TITLE-ABS ( africa OR algeria OR angola OR benin OR botswana OR "Burkina Faso" OR burundi OR "Cabo Verde" OR cameroon OR "Central African Republic" OR chad OR comoros OR congo OR "Democratic Republic of the Congo" OR "Cote d&apos;Ivoire" OR djibouti OR egypt OR "Equatorial Guinea" OR eritrea OR eswatini OR ethiopia OR gabon OR gambia OR ghana OR guinea OR "Guinea-Bissau" OR kenya OR lesotho OR liberia OR libya OR madagascar OR malawi OR mali OR mauritania OR mauritius OR morocco OR mozambique OR namibia OR niger OR nigeria OR rwanda OR "Sao Tome and Principe" OR senegal OR seychelles OR "Sierra Leone" OR somalia OR "South Africa" OR "South Sudan" OR sudan OR tanzania OR togo OR tunisia OR uganda OR zambia OR zimbabwe OR "Northern Africa" OR "Middle Africa" OR "Eastern Africa" OR "Western Africa" ) AND PUBYEAR > 1999 AND PUBYEAR < 2025 AND NOT ( "organ traffic*" ) AND ( EXCLUDE ( DOCTYPE , "ed" ) ) AND ( LIMIT-TO ( LANGUAGE , "English" ) OR LIMIT-TO ( LANGUAGE , "French" ) OR LIMIT-TO ( LANGUAGE , "Spanish" ) ) |
